# Strychnia in Impaired Spinal Energy

**Published:** 1853-09

**Authors:** 


*Strychnia in Impaired Spinal Energy.*—Dr. Marshall Hall believes that this condition is due to causes of nervous exhaustion, such as excessive study, muscular effort, and sexual indulgence, and has found strychnia useful in correcting it. He gives minute doses thrice daily, for many months, in the midst of meals. The following formula may be used:

℞ strychniæ acetatis, gr. j.

acidi acetici, gtt. xv.

alchoholis, ℥ij.

aquæ destillatæ, ℥vj. M.

Dose, ten drops, containing one-fiftieth of a grain.

The remedy acts on the spinal marrow, and is counter-indicated in cases of *irritation* of the nervous center and of spasm.—*Lancet.*

